# The potential impact of plain packaging of cigarette products among Brazilian young women: an experimental study

**DOI:** 10.1186/1471-2458-12-737

**Published:** 2012-09-04

**Authors:** Christine M White, David Hammond, James F Thrasher, Geoffrey T Fong

**Affiliations:** 1 School of Public Health & Health Systems, University of Waterloo, 200 University Avenue West, Waterloo, ON N2L 3G1, Canada; 2 Department of Health Promotion, Education and Behavior, Arnold School of Public Health, University of South Carolina, 800 Sumter Street, Room 215, Columbia, SC, 29208, USA; 3 Departamento de Investigaciones sobre Tabaco, Centro de Investigación en Salud Poblacional, Instituto Nacional de Salud Pública, Cuernavaca, Mexico; 4 Department of Psychology, University of Waterloo, 200 University Avenue. West, Waterloo, ON, N2L 3G1, Canada; 5 Ontario Institute for Cancer Research, Toronto, ON, Canada

**Keywords:** Tobacco, Packaging, Marketing, Health policy

## Abstract

**Background:**

Tobacco use is responsible for 5.4 million deaths every year worldwide and is a leading cause of preventable death. The burden of these deaths is rapidly shifting to low and middle-income countries, such as Brazil. Brazil has prohibited most forms of tobacco advertising; however, the cigarette pack remains a primary source of marketing. The current study examined how tobacco packaging influences brand appeal and perceptions of health risk among young women in Brazil.

**Methods:**

A between-subjects experiment was conducted in which 640 Brazilian women aged 16–26 participated in an online survey. Participants were randomized to view 10 cigarette packages according to one of three experimental conditions: standard branded packages, the same packs without brand imagery (“plain packaging”), or the same packs without brand imagery or descriptors (e.g., flavors). Participants rated packages on perceived appeal, taste, health risk, smoothness, and smoker attributes. Finally, participants were shown a range of branded and plain packs from which they could select one as a free gift, which constituted a behavioral measure of appeal.

**Results:**

Branded packs were rated as significantly more appealing, better tasting, and smoother on the throat than plain packs. Branded packs were also associated with a greater number of positive smoker attributes including style and sophistication, and were perceived as more likely to be smoked by females than the plain packs. Removing descriptors from the plain packs further decreased the ratings of appeal, taste and smoothness, and also reduced associations with positive attributes. In the pack offer, participants were three times more likely to select branded packs than plain packs.

**Conclusions:**

Plain packaging and removal of descriptors may reduce the appeal of smoking for youth and young adults, and consequently reduce smoking susceptibility. Overall, the findings provide support for plain packaging regulations, such as those in Australia.

## Background

Tobacco use is the world’s leading cause of preventable death [1]. The global burden of tobacco use is rapidly shifting from high-income “Western” countries to low and middle income countries [2]. Brazil – an upper middle-income country – is a particularly important country for global tobacco control given that it accounts for the seventh largest number of smokers in the world, approximately 25 million smokers [1,3]. Overall, 17.2% of the Brazilian population aged 15 years or older are current smokers [3]. As in most other countries, the vast majority of smokers in Brazil start before age 19, with women typically starting earlier than men [3]. It is estimated that, in 2008, 13.1% of women aged 15 or older (9.8 million) in Brazil were smokers, including 6.4% of women aged 15 to 24 [3].

Brazil is widely regarded as an international leader in tobacco control [4]. The government has prohibited most forms of advertising, including bans on national television, radio, and print media [1]. In the face of these restrictions, the cigarette package itself has become one of the primary tobacco marketing tools used by the industry. Indeed, internal tobacco industry documents underscore this point: “*Our final communication vehicle with our smoker is the pack itself. In the absence of any other marketing messages, our packaging…is the sole communicator of our brand essence”*[5].

Research has consistently demonstrated that branding on cigarette packages is targeted towards young adults and women, and may increase the appeal of smoking [6-8]. Packaging can convey product characteristics and help “position” a brand so that a particular image and identity is promoted. Common brand images targeted at woman include social status, glamour, slimness, and femininity [9]. Use of traditionally female colors on the package or as brand descriptors may also be used to target women and portray smoking as feminine and stylish [10-12]. Similarly, descriptors such as “slim” and the use of thin “lip-stick” shaped packs can be used to appeal to young women’s concerns about body weight and the perceived relationship between cigarette smoking and thinness [10].

Cigarette taste and flavor also influence cigarette appeal and make the initial experience of smoking less aversive to youth [13]. Brands targeted at youth are typically marketed as smoother and less harsh, and include flavors that may be more palatable such as mint, or strawberry. The names of these flavors are often featured in the package descriptors and may increase smoking appeal [13].

Brand descriptors and imagery on cigarette packaging can falsely reassure consumers about the potential risks of their products. Studies have shown that many smokers mistakenly believe that cigarettes labeled as “light” or “mild” actually deliver less tar and are less harmful to smokers, and consequently are “healthier” than regular cigarettes [14,15]. Although Brazil banned the use of these misleading descriptors in 2001, a number of brands use alternative terms such as “fresh” or references to lighter colors such as “gold” or “silver” [3]. Elements such as the pack color and shape can also reinforce false beliefs among smokers [7,16].

Plain packaging has been recommended by the World Health Organization (WHO) Framework Convention on Tobacco Control (FCTC) as a component of marketing restrictions [17]. Plain packaging regulations would prohibit logos, colors, and images from appearing on packages. Manufacturers would only be permitted to print the brand name and descriptors in a standard font and size against a standard background color. In December 2012, Australia will become the first country in the world to introduce plain packaging [18].

Research in “Western” countries has indicated that plain packaging has the potential to impact youth smoking perceptions and behaviors. Youth perceive plain packages as less appealing and have more negative expectations of cigarette taste [19,20]. They are also less likely to associate brands in plain packages with favorable personality traits such as being trendy and sociable [19]. Additionally, individuals shown plain packages are less likely to falsely believe that certain brands are less harmful, deliver less tar, or are easier to quit [21]. However, the effect of packaging has yet to be systematically tested in other markets, including Latin American countries such as Brazil.

The current study sought to experimentally manipulate pack branding to examine the impact of cigarette pack design on female youth in Brazil. Specifically, this study examined the effect of color variations, imagery, and brand descriptors, as well as the removal of these elements (i.e., plain packaging) on perceptions of brand appeal, taste, health risk, smoothness, and smoker attributes. Conducting the study in Brazil provided an opportunity to examine the impact of packaging in one of the largest and most important global tobacco markets.

## Methods

### Participants and recruitment

Participants consisted of 640 young women (16–26 years) from Brazil, including smokers and non-smokers. Female youth and young adults were chosen because this age is a critical period for smoking initiation and female youth are thought to be especially influenced by branding [11]. Participants were recruited from an online panel through Global Market Insite, Inc. (GMI), a commercial market research company with a panel reach of over 350,000 Brazilians. The panel included residents living in any region of Brazil. While the sample may not have necessarily been representative of the entire female young adult population in Brazil, the sample does represent a national heterogeneous group of young women. Additional information on the GMI panel is available online (http://www.gmi-mr.com). Panel members were invited to participate in the online survey via e-mail, but were not informed about the purpose of the study. Upon survey completion, participants were given remuneration of approximately R$2.80 ($1.60 USD). The study received ethics clearance from the Office of Research Ethics at the University of Waterloo, Ontario, Canada, and all participants provided consent prior to completing the survey, in accordance with ethics requirements at the University of Waterloo.

### Protocol

After providing consent, participants completed a brief background survey that included key questions on smoking behaviors and socio-demographics. Participants were then randomly assigned to one of three experimental conditions: 1) “*branded*” packages; 2) *“plain”* packages: the same packages with all brand imagery removed, including colors and graphics, but with brand descriptors maintained; or 3) “*plain-no descriptors*” packages: the same packages with both descriptors and imagery removed.

In each condition, participants were shown color images of 10 individual cigarette packages, one at a time in a random order and were asked to rate each pack “compared to other cigarette brands you can buy in stores” on four *brand ratings* and five *smoker image* questions (described below). At the end of the study, participants completed a behavioral task in which they were asked to select which, if any, cigarette pack they would like to be sent upon conclusion of the study (note: packages were not actually sent).

### Cigarette packages

Ten female-oriented brands were selected for the current study, including four brands sold in Brazil *(Virginia Slims Silver, Dunhill Carlton – Carlton Mint Blend, Vogue Bleue and Marlboro Gold Original)*, and six other leading international cigarette brands *(Peel Sweet Melon, John Player Special Pink, Benson & Hedges Superslims Park Avenue, DJ Mix Strawberry Flavor, Silk Cut Superslims Menthol, and Capri Baunilha).* Brands were purposively selected to feature different color descriptors (*silver, gold, bleue, and pink*) and flavor descriptors (*baunilha/vanilla, strawberry, mint, sweet melon, and menthol*), as well as other descriptors such as *superslims*. Packages that featured “traditional” female color schemes, including the use of pink, light green, light blue, and white, as well as smaller pack shapes, were also selected.

**Figure 1:**
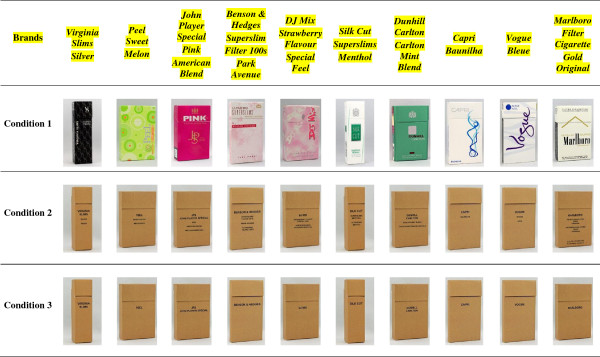
Cigarette package images by experimental condition.

Portuguese text was digitally added to packages with English-only text to ensure that participants who could only read Portuguese would be able to distinguish the packages in the *plain* condition and the *plain-no descriptors* condition. Since pictorial health warning labels are only shown on the back side of the package in Brazil, these were not visible to the participants in any of the images shown. The order in which the packages were viewed was counter-balanced across participants. See Figure 1 for pack images shown in each of the three conditions.

### Measures

#### Socio-demographic variables and moderators

Socio-demographic measures included age, education level, ethnicity, and smoking status, and were assessed using previously validated measures adapted from the International Cigarette Packaging Study surveys and the Brazil National International Tobacco Control (ITC) survey. *Education* was categorized as “low” (completing ensino medio/high school or less), “medium” (some ensino superior/post-secondary school), and “high” (completed ensino superior/post-secondary school, some graduate school or completed graduate school). Participants were asked to identify their *ethnicity* as white, black, Asian, pardo (mixed ancestry), Indian, and/or other, as per the categories listed in the Brazilian census. Participants could select all categories that applied. Ethnicity was then categorized as “white” (respondents who only identified themselves as white), “pardo” (respondents who only identified themselves as pardo/mixed ancestry) and “other” (respondents who identified themselves as black, Asian, Indian, other, or multi-racial [chose multiple categories]). Participants were asked how often they smoked cigarettes in the last 30 days. Smokers were defined as respondents who reported smoking daily (every day); weekly (at least once a week, but not every day), or monthly (at least once in the last 30 days, but not every week). Non-smokers were defined as respondents who reported smoking less than monthly (did not smoke in the last 30 days).

#### Brand ratings

Participants were asked to rate each of the 10 packages “compared to other brands you can buy in stores” on four measures: 1) brand appeal (“compared to other brands you can buy in stores, how appealing is this brand of cigarettes?”); 2) perceived taste (“…how do you think these cigarettes would taste?”); 3) health risk (“…would these cigarettes be… less/more harmful”); and 4) smoothness (“…how smooth do you think these cigarettes would be on your throat?”). Responses were provided on a 5-point Likert scale (e.g., 1 = “A lot more appealing” to 5 = “A lot less appealing”) and were subsequently re-coded into a binary variable as either a 1 (“a little” / “a lot more appealing”) or 0 (“a little” / “a lot less appealing” and “no difference”). A summary index rating was created for each of the four brand rating measures, by summing scores across the 10 packages to yield a score between 0 and 10, where the number corresponded to the total number of packs rated as more appealing/better taste/less harmful/smoother on the throat.

#### Smoker image ratings

For each package, respondents were asked to identify the typical smoker of each pack by answering the question, “In your opinion, is someone who smokes this brand regularly more likely to be…” for five characteristics: female/male, stylish/not stylish, popular/not popular, sophisticated/not sophisticated, and slim/overweight. For each set of characteristics, respondents were able to choose either trait, or “no difference”. Responses were scored as a “1” if the respondent selected the more desirable trait (female, stylish, popular, sophisticated, or slim) and “0” if the respondent selected the less desirable trait (e.g., male, not stylish, not popular, not sophisticated, or overweight), “no difference,” or “don’t know”. A summary index was created for each of the five characteristics by summing scores across the 10 packages to yield a score between 0 and 10, where the number corresponded to the total number of times the desirable trait was endorsed. A single overall “positive smoker image index” was also created by calculating the mean of these five index scores (range = 0 to 10).

#### Pack selection task

Prior to the conclusion of the study, participants were told that as a thank-you gift for completing the survey, they could, if they wished, select a pack they would like to be sent from a choice of four cigarette packages shown on the screen. Participants were shown four packages: two *branded* packages and two *plain* packages, regardless of the condition they were assigned to earlier in the survey. Packs were drawn at random from those displayed previously. The participants had the option to select one of the four packages shown, or select an “I do not wish to receive a package” option, prominently displayed on the screen. Immediately after making their selection, the participants were informed that no packages would be mailed as the investigators did not want to endorse smoking.

All key measures including those for the brand ratings, smoker image ratings and pack selection task were adapted from previous research [16] and were translated into Portuguese by two independent bilingual translators. Cognitive pre-testing of the survey was conducted to ensure that the translated questions conveyed the intended meaning in a clear manner that minimized response error [22].

### Analysis

Chi square tests were used to assess differences in key socio-demographic factors between experimental conditions. Logistic regression models were used to examine the effect of the experimental conditions for single packages on the four brand attributes, and to examine the extent to which participants selected a pack (*branded* or *plain*) in the pack selection task. Linear regression models were used to examine the effect of the experimental conditions on each of the four brand attribute and six smoker image index variables, including the overall “positive smoker image index”. All beta (β) and odds ratio (OR) values from regression models are adjusted for age, education, ethnicity, and smoking status. All analyses were conducted in SPSS version 19.0.

## Results

### Sample characteristics

Table 1 shows the sample characteristics. Other than for ethnicity (*χ*^2^ = 11.2, p = 0.025), there were no statistically significant differences in the characteristics of participants across the three experimental conditions.

**Table 1:** Sample characteristics (n = 640)

**Characteristic**	**Overall**	**Branded**	**Plain**	**Plain - no descriptor (n = 218)**	**Differences between groups**
	**(n = 640)**	**(n = 214)**	**(n = 208)**		
**Age**					
Mean	22.4	22.4	22.4	22.4	F = 0.1
	SD = 2.4	SD = 2.3	SD = 2.4	SD = 2.5	p = 0.905
**Education level**					
Low	21.9% (140)	18.8% (40)	21.6% (45)	25.1% (55)	*χ*^2^ = 3.3
Moderate	48.3% (309)	48.8% (104)	50.5% (105)	45.7% (100)	p = 0.512
High	29.8% (191)	32.4% (69)	27.9% (58)	29.2% (64)	
Ethnicity*					
White	62.9% (401)	54.7% (116)	65.2% (135)	68.5% (150)	*χ*^2^ = 11.2
“Pardo”	25.2% (161)	32.5% (69)	23.7% (49)	19.6% (43)	p = 0.025
Other	11.9% (76)	12.7% (27)	11.1% (23)	11.9% (26)	
**Smoking status**					
Smoker	28.4% (182)	28.2% (60)	27.9% (58)	29.2% (64)	*χ*^2^ = 0.1
Non-smoker	71.6% (458)	71.8% (153)	72.1% (150)	70.8% (155)	p = 0.949
**Smoking frequency**^a^					
Daily	39.0% (71)	43.3% (26)	41.4% (24)	32.8% (21)	*χ*^2^ = 2.0
Weekly	24.2% (44)	20.0% (12)	24.1% (14)	28.1% (18)	p = 0.732
Monthly	36.8% (67)	36.7% (22)	34.5% (20)	39.1% (25)	
**Cigarettes per day**^a^					
Mean	10.8	9.8	10.5	12.3	F = 0.6
	SD = 7.6	SD = 5.8	SD = 8.8	SD = 8.2	p = 0.537
**Quit intentions**^a^					
Within the next month	12.9% (18)	16.7% (8)	8.3% (4)	14.0% (6)	*χ*^2^ = 4.5
Within the next 6 months	18.0% (25)	14.6% (7)	22.9% (11)	16.3% (7)	p = 0.609
Sometime in the future	53.2% (74)	47.9% (23)	58.3% (28)	53.5% (23)	
Not planning to quit	15.8% (22)	20.8% (10)	10.4% (5)	16.3% (7)	

SD, standard deviation.

*Statistically significant difference between experimental conditions (p < 0.05).

^a^Among current smokers only.

### Brand ratings

#### Appeal ratings

Table 2 shows brand appeal ratings for the 10 individual packs. The highest appeal ratings were given for the *branded Virginia Slims Silver, Peel Sweet Melon*, and *John Player Special Pink* packs. A linear regression was conducted using an index score for brand appeal that combined all ten packs to examine overall differences in appeal between the experimental conditions, adjusting for age, education, ethnicity, and smoking status. A significant main effect of condition was found (F = 43.1, p < 0.001), where packs in the *branded* condition (mean = 6.0) were rated as significantly more appealing than packs in the *plain* condition (mean = 4.3, β = 1.64, p < 0.001), and *plain-no descriptors* condition (mean = 3.4, β = 2.53, p < 0.001). The *plain* packs were also given significantly higher appeal ratings than the *plain, no descriptor* packs (β = 0.89, p = 0.002). None of the covariates had a statistically significant association with appeal.

**Table 2:** Brand ratings for individual cigarette packages by experimental condition (n = 640)

	***Virginia Slims Silver***	***Peel Sweet Melon***	***John Player Special Pink American Blend***	***Benson & Hedges Superslims Filter 100s Park Avenue***	***DJ Mix Strawberry Flavour Special Feel***	***Silk Cut Superslims Menthol***	***Dunhill Carlton Carlton Mint Blend***	***Capri Baunilha***	***Vogue Bleue***	***Marlboro Filter Cigarettes Gold Original***	
											**INDEX SCORE**
**“A little” or “A lot” MORE APPEALING than other brands** (% agree)											(Mean score)
**Standard**	77.1%^a^	72.5%^a^	71.6%^a^	69.5%^a^	68.9%^a^	58.1%^ab^	50.0%^a^	49.5%^a^	45.9%^a^	23.4%^a^	6.0^a^
**Plain**	48.7%^b^	39.9%^b^	32.8%^b^	33.9%^b^	51.1%^b^	60.6%^a^	40.2%^b^	45.9%^a^	39.1%^a^	39.8%^b^	4.3^b^
**Plain-no desc.**	49.5%^b^	13.8%^c^	29.1%^b^	29.1%^b^	14.6%^c^	50.5%^b^	33.8%^b^	27.5%^b^	59.7%^b^	38.5%^b^	3.4^c^
**“A little” or “A lot” BETTER TASTE than other brands** (% agree)											(Mean score)
**Standard**	39.6%^a^	65.5%^a^	45.7%^a^	56.8%^a^	66.3%^a^	60.6%^a^	45.3%^a^	57.7%^a^	25.3%^a^	24.5%^a^	4.9^a^
**Plain**	25.4%^b^	50.0%^b^	23.7%^b^	20.5%^b^	55.8%^b^	59.1%^a^	50.3%^a^	55.2%^a^	26.6%^a^	29.8%^a^	3.9^b^
**Plain-no desc.**	35.0%^a^	9.7%^c^	21.5%^b^	20.6%^b^	12.0%^c^	32.8%^b^	27.0%^b^	18.7%^b^	33.2%^a^	30.8%^a^	2.3^c^
**“A little” or “A lot” LESS HEALTH RISK than other brands** (% agree)											(Mean score)
**Standard**	8.8%^a^	22.5%^a^	10.3%^a^	24.0%^a^	14.1%^a^	18.0%^a^	9.3%^a^	13.9%^a^	12.5%^a^	11.4%^a^	1.5^a^
**Plain**	9.7%^a^	10.7%^b^	7.4%^a^	17.9%^ab^	9.8%^a^	10.9%^a^	11.5%^a^	14.5%^a^	8.5%^a^	16.1%^a^	1.1^a^
**Plain-no desc.**	11.6%^a^	14.1%^ab^	11.0%^a^	10.6%^b^	11.5%^a^	14.1%^a^	8.9%^a^	9.0%^a^	10.6%^a^	10.8%^a^	1.2^a^
**“A little” or “A lot” MORE SMOOTH than other brands** (% agree)											(Mean score)
**Standard**	23.3%^a^	56.5%^a^	35.0%^a^	46.2%^a^	54.2%^a^	51.5%^a^	40.5%^a^	50.5%^a^	26.3%^ab^	21.1%^a^	4.1^a^
**Plain**	22.0%^a^	41.1%^b^	16.8%^b^	25.9%^b^	42.1%^b^	43.8%^a^	36.0%^a^	45.1%^a^	19.5%^a^	25.9%^a^	3.1^b^
**Plain-no desc.**	29.1%^a^	12.2%^c^	11.6%^b^	11.1%^c^	7.5%^c^	19.8%^b^	16.2%^b^	19.5%^b^	29.1%^b^	13.4%^b^	1.6^c^

*Letters are used to indicate statistical significance between values in the same column based on logistic regression models for the single packages, and linear regression models for the index scores. Values with different letters are significantly different at the p < 0.05 level. All regression models were adjusted for age, education, ethnicity, and smoking status.

Mean index scores were created for each measure by summing the number of times “A little” or “A lot” was selected across the 10 brands (range: 1 to 10).

#### Pack taste ratings

The four highest ratings of taste were given to *branded* packages with flavor descriptors: *DJ Mix Strawberry Flavor*, *Peel Sweet Melon*, *Silk Cut Menthol,* and *Capri Baunilha*. A linear regression model was conducted using the taste index variable that combined all ten packs, and examined differences in taste ratings across experimental conditions and socio-demographic predictors. A significant main effect of condition was found (F = 45.7, p < 0.001), such that the *branded* packs (mean = 4.9) were given higher taste ratings than the *plain* packs (mean = 3.9, β = 1.01, p < 0.001), and the *plain, no descriptor* packs (mean = 2.3, β = 2.62, p < 0.001). In addition, packs in the *plain* condition were given significantly higher taste ratings than the packs in the *plain-no descriptors* condition (β = 1.60, p < 0.001). Furthermore, smokers were significantly more likely to rate packs as better tasting than non-smokers (β = 0.88, p < 0.001).

#### Health risk ratings

Table 2 shows the health risk ratings for each of the ten individual packs. Overall, 42.6% of respondents reported that at least one of the ten brands would be “less harmful” than other brands. In a linear regression model using the health risk index variable that combined all ten packs, no significant main effect of condition was observed (F = 1.6, p = 0.207). Younger participants (β = 0.11, p = 0.007) and smokers (β = 0.45, p = 0.019) were significantly more likely to rate packs as “less harmful”. In contrast, participants who identified their ethnicity or race as “white” were less likely than people identifying as “other” to rate packs as “less harmful” (β = −0.74, p = 0.008).

#### Smoothness ratings

Similar to the taste ratings, the top four smoothness ratings were given for *branded* packages with flavor descriptors: *Peel Sweet Melon*, *DJ Mix Strawberry Flavor*, *Silk Cut Menthol*, and *Capri Baunilha*. In a linear regression model using the smoothness index score, a significant main effect of condition was observed (F = 42.1, p < 0.001), where the *branded* packs (mean = 4.1) were given higher smoothness ratings than the *plain* packs (mean = 3.1, β = 1.01, p < 0.001) and *plain-no descriptors* packs (mean = 1.6, β = 2.50, p < 0.001). Packs in the *plain* condition were also given significantly higher smoothness ratings than the packs in the *plain-no descriptors* condition (β = 1.49, p < 0.001). Again, smokers were significantly more likely to rate packs as “more smooth” than non-smokers (β = 0.79, p = 0.001).

#### Differences by brand familiarity

Paired sample t-tests were conducted to compare the mean ratings for the *branded* domestic packs versus *branded* international packs on each of the brand rating measures (appeal, taste, harm and smoothness). Respondents were significantly more likely to rate international brands as more appealing (p < 0.001), better tasting (p < 0.001) and smoother on the throat (p < 0.001) than domestic brands. In contrast, respondents were significantly more likely to rate domestic brands as less harmful (p = 0.001) than international brands.

### Smoker image ratings

Participants were asked to rate each pack along five smoker image traits. Table 3 shows the mean number of packs endorsed as the more desirable/positive trait across the ten packs viewed. *Plain* packs and *plain-no descriptors* packs both received significantly fewer positive ratings than *branded* packs for four of the five traits.

**Table 3:** Index scores of perceived smoker traits by experimental condition (n = 623)

	**Female**	**Stylish**	**Popular**	**Sophisticated**	**Slim**	**Smoker trait index**
**Mean score (SD)**						
**Branded**	4.7^a^	4.6^a^	3.0^a^	4.1^a^	2.3^a^	3.8^a^
	(1.7)	(2.7)	(2.5)	(2.8)	(2.6)	(2.0)
**Plain**	4.1^b^	3.7^b^	2.5^b^	3.4^b^	2.1^a^	3.2^b^
	(2.2)	(2.6)	(2.5)	(2.6)	(2.6)	(2.1)
**Plain-no descriptors**	2.7^c^	3.0^c^	2.4^b^	2.7^c^	1.9^a^	2.7^c^
	(1.8)	(2.5)	(2.5)	(2.5)	(2.3)	(2.0)

SD, standard deviation.

*Letters are used to indicate statistical significance between values in the same column based on logistic regression models for the single packages, and linear regression models for the index scores. Values with different letters are significantly different at the p < 0.05 level.

All regression models were adjusted for age, education, ethnicity, and smoking status.

Mean index scores were created by summing the number of desirable traits endorsed across the 10 brands (range: 1 to 10).

In a linear regression in which all five of the smoker traits predicting “positive” image ratings score were combined into a single smoker image index, where higher scores indicated more positive traits, a significant main effect of condition was found (F = 16.0, p < 0.001), such that packs in the *branded* condition (mean = 3.84) were given higher “positive” trait scores than their counterparts in the *plain* (mean = 3.22, β = 0.62, p = 0.003), and *plain-no descriptors* (mean = 2.67, β = 1.20, p < 0.001) conditions. Packs in the *plain* condition were also given higher “positive” trait scores than the packs in the *plain-no descriptors* condition (β = 0.57, p = 0.005). Additionally, smokers were more likely to rate the packs as having a positive trait than non-smokers (β = 1.05, p < 0.001).

### Pack selection task

At the end of the survey, participants were offered a pack of cigarettes and were told they could either chose not to receive a pack, or could select a pack from one of four options (two branded packs, and two plain packs with descriptors). The packs were randomly selected from the ten brands previously viewed. Overall, 52.1% (n = 325) accepted the pack offer and selected a package. Smokers were significantly more likely to select a pack than non-smokers (OR = 9.11, 95% CI = 5.8-14.3, p < 0.001), with 84.5% of smokers (n = 153) and 38.8% of non-smokers (n = 172) accepting a pack. In total, 39.6% of participants chose a *branded* package and 12.5% chose a *plain* package. The most frequently chosen packages were the *branded DJ Mix Strawberry Flavor*, *John Players Special Pink*, *Benson & Hedges Superslims*, *Virginia Slims Silver*, and *Peel Sweet Melon* pack. The *plain* packages that were chosen most frequently all contained flavor descriptors. In a logistic regression of sample characteristics predicting whether participants would chose a *branded* pack or *plain* pack, it was found that smokers were less likely to select a *branded* pack than non-smokers (OR = 0.35, 95%CI = 0.20-0.61, p < 0.001). Experimental condition was also significant, such that participants in the *plain-nondescriptors* condition were more likely to select a *branded* pack than participants in the *branded* condition (OR = 2.25, 95% CI = 1.14-4.41, p = 0.019).

## Discussion

To our knowledge, this is the first study to examine the impact of cigarette package design in Latin America, and one of the few studies in any country to focus on young women. The findings indicate that removing color and brand descriptors significantly reduced brand appeal. The greatest decreases in brand appeal were observed when both brand imagery and descriptors were removed from packages. These findings are consistent with previous studies suggesting that when cigarette packages are stripped of their color, imagery and fonts, youth find the packs less appealing [7,16,19,23].

In the *branded* condition, single packs with female-oriented color schemes (e.g., pinks, light green) and designs (e.g., the black *Virginia Slims* pack) were generally rated more favorably than packs that appeared more gender neutral (e.g., the white *Marlboro Original Gold* pack). These findings are consistent with research on industry documents that suggest that marketers advertise ‘female’ brands of cigarettes as a means of satisfying their needs such as peer belonging, self-confidence, and female camaraderie [8]. The “slim” and “lipstick” shaped packages were also rated relatively favorably, even in the *plain* conditions. In fact, these narrower packages were rated as "more appealing" by the largest proportion of people across all 10 packs in the *plain-no descriptors* condition. It is unclear whether this was due to the “slim” pack shape or the overtly feminine brand family names that remained on packs (e.g., *Silk Cut*, and *Virginia Slims*). Future research should consider experimentally manipulating pack shape and size. The present findings are consistent with past research and suggest that regulators may need to consider restricting pack size and shape if they wish to reduce brand appeal among young women [24].

The findings also highlight the potential appeal of “brand family names” such as *Vogue*. Indeed, the *plain* pack with only the name *Vogue* was rated as more appealing by a greater proportion of women than the *Vogue* pack that also featured the descriptor “*bleue*” (60% versus 39%, respectively). If plain packaging is implemented, one might expect the tobacco industry to create not only more appealing brand descriptors, but more alluring brand names, including those with pre-existing associations with glamour and fashion. Future studies could monitor the extent to which tobacco companies alter brand names and the use of brand descriptors following implementation of plain packaging regulations in Australia.

The removal of imagery and descriptors also influenced perceptions of taste. Across the brands, the greatest decreases in perceived taste were observed when both the imagery and the descriptors were removed. This was especially true for the five flavored cigarette packages. While removing the brand imagery significantly reduced taste ratings for two of the five flavored brands, removing both the imagery and the descriptors significantly reduced taste ratings for all five flavored brands. This finding indicates that plain packaging and greater restrictions on descriptors—or even prohibiting flavored cigarettes— could reduce positive perceptions of taste, and potentially undermine an individual’s desire to smoke [25]. These results are particularly notable given that Brazil recently announced a ban on flavor additives, including menthol.

The findings on perceived smoothness were similar to those for brand appeal and taste. Across the brands, perceived smoothness was lowest for *plain-no descriptors* packs. Removing the brand imagery significantly reduced smoothness ratings for four packages –the four packages that were also rated most frequently as “female”. This may indicate that consumers associate feminine packs with being less harsh or smoother on the throat. Previous research indicates that perceived smoothness is an important predictor for smoking initiation among youth and is strongly correlated with perceptions of risk [21,26,27]. Brands perceived as smooth may promote experimentation or smoking initiation by reducing harshness and irritation [26].

It was initially hypothesized that plain packaging would reduce the misconception that particular brands were less harmful than others; however, no significant differences between conditions were observed. This difference may not have been observed because of a “floor effect” in the health risk ratings. For the majority of *branded* packages, less than 15% of the participants rated the packs as “less harmful”; therefore, there was little room for this proportion to significantly decrease across the other conditions. The presence of a floor effect may indicate that the tobacco control messages in Brazil, including large pictorial health warnings, may have been effective in communicating that all cigarette brands are equally harmful, and that there are no “safe” cigarettes [28,29]. The effect of packaging on perceptions of risk may be different in other low-and middle-income countries with lower awareness about the health effects of smoking. Previous research suggests that plain packaging can increase attention to health warnings [21,30]. Cigarette packages shown in the current study, however, did not display a pictorial health warning on the package front, as per existing regulations in Brazil. If health warnings were visible during the study, this may have led to differences in health ratings between the *plain* packages and *branded* packages.

Plain packaging reduced the likelihood that young women would associate particular packs with attributes such as being female, stylish, and/or sophisticated. These findings add to the evidence that cigarette packaging acts as a type of “lifestyle” advertising [16,19].

The pack selection task provided additional evidence that *branded* packaging was more desirable than *plain* packaging among female youth. Participants were three times more likely to select a *branded* pack than a *plain* pack, consistent with previous research [16]. The findings also demonstrate that, as one might expect, *plain* packages with more engaging brand names and descriptors were more likely to be selected. The two *plain* packages chosen most frequently had flavor descriptors (*DJ Mix Strawberry Flavor* and *Peel Sweet Melon*). This highlights the appeal of flavored cigarettes to youth, and provides further support for a ban on flavored cigarettes [13].

### Strengths and limitations

Participants in this study were not recruited through random sampling and were limited to individuals with internet access. In 2011, Brazil had an internet penetration of 41%, or almost 76 million people [31]. Individuals with internet access likely have a higher degree of education and literacy than the general population. In addition, the self-reported smoking prevalence in our sample (28.4%) was higher than national smoking prevalence estimates for young women. Therefore, the findings may not generalize to the broader population of female youth in Brazil.

Some degree of self-selection bias was also likely given that participants self-selected into the study by enrolling in GMI’s participant panel; however, participants were unaware of the topic of study when beginning the survey. In addition, cigarette branding and industry marketing is national in scope and varies very little across regions of Brazil [32]; therefore, one would expect little systematic differences in tobacco/branding exposure between the current sample and the general population. Furthermore, the between-subjects experimental design and randomization of participants to experimental conditions should ensure that any sampling biases were equally distributed across groups and therefore could not account for differences found among conditions.

The extent to which the current findings apply to other Latin American countries is unclear due to cultural differences between countries, for example, in terms of what packaging elements the participants find appealing, or stylish. Differences by country in the implementation of other tobacco control policies, such as advertising bans and the use of pictorial health warnings on packaging could also influence perceptions. However, pack design and other tobacco marketing practices are often similar across countries, and the findings from this study were similar to those obtained in studies conducted in many Western countries [7,16].

In this study, participants viewed images of cigarette packages, rather than observing packs directly. It is inherently difficult for packaging studies to replicate “naturalistic” exposure to packaging as it occurs in real-life. Multiple factors such as package display, advertising, peer influences, and even the weight and feel of the packs may enhance the influence of package design. Consequently, the findings may underestimate the impact of package design.

The inclusion of the pack selection task allowed brand appeal to be assessed using not only a 5-point Likert scale, but also as a behavioral measure. The order in which the packs were selected in terms of frequency was generally consistent with the ratings of appeal in the individual pack rating section. This consistency provides some evidence of the validity of the pack rating questions as proxies for consumer behavior.

## Conclusions

This study is one of the first to experimentally examine young women’s perceptions of plain packaging in Latin America. The results are comparable to packaging studies in other countries using similar study protocols, which highlights common marketing practices used across global markets. Overall, the findings support the recommendations for plain packaging in the WHO Framework Convention on Tobacco Control and Australia's recent plain packaging regulations.

## Competing interests

The authors declare that they have no competing interests.

## Authors' contributions

CMW, DH, JFT, and GTF contributed to the design of the study, and manuscript preparation. CMW also performed the data analysis and drafted the manuscript. All authors read and approved the final manuscript.

## Pre-publication history

The pre-publication history for this paper can be accessed here:

http://www.biomedcentral.com/1471-2458/12/737/prepub
